# Prognostic Characteristics of Immune-Related Genes and the Related Regulatory Axis in Patients With Stage N+M0 Breast Cancer

**DOI:** 10.3389/fonc.2022.878219

**Published:** 2022-06-16

**Authors:** Chonglin Tian, Yongsheng Wang, Xianrang Song

**Affiliations:** ^1^ Department of Burn and Plastic Surgery, Shandong Provincial Hospital Affiliated to Shandong First Medical University, Jinan, China; ^2^ Department of Burn and Plastic Surgery, Shandong Provincial Hospital, Cheeloo College of Medicine, Shandong University, Jinan, China; ^3^ Shandong Cancer Hospital and Institute, Shandong First Medical University and Shandong Academy of Medical Sciences, Jinan, China

**Keywords:** immune-related genes, risk score, BRCA, lymph node metastasis, prognosis

## Abstract

Breast cancer (BRCA) has the highest incidence rate among female tumours. The function of the immune system affects treatment efficacy and prognosis in patients with BRCA. However, the exact role of immune-related genes (IRGs) in stage N+M0 BRCA is unknown. We constructed a predictive risk scoring model with five IRGs (*CDH1*, *FGFR3*, *INHBA*, *S100B*, and *SCG2*) based on the clinical, mutation, and RNA sequencing data of individuals with stage N+M0 BRCA sourced from The Cancer Genome Atlas. Results from the Shandong Cancer Hospital and Institute validation cohort suggested that regardless of clinical stage, tumour size, or the number of lymph node metastases, this model was able to reliably discriminate low-risk patients from high-risk ones and assess the prognosis of patients with stage N+M0 BRCA, and low-risk patients could benefit more from immunotherapy than high-risk patients. In addition, significant inter-group variations in immunocyte infiltration and the tumour microenvironment were observed. Moreover, risk score and age were found to be independent factors in multivariate COX regression analysis, which influenced the outcome of patients with stage N+M0 BRCA. Based on the above findings, we plotted a prognostic nomogram. Finally, we constructed a lncRNA KCNQ1OT1-LINC00665-TUG1/miR-9-5p/CDH1 regulatory axis of the ceRNA network to explore the mechanism of BRCA progression. In summary, we conducted a systemic and extensive bioinformatics investigation and established an IRG-based prognostic scoring model. Finally, we constructed a ceRNA regulatory axis that might play a significant role in BRCA development. More research is required to confirm this result. Scoring system-based patient grouping can help predict the outcome of patients with stage N+M0 BRCA more effectively and determine their sensitivity to immunotherapies, which will aid the development of personalised therapeutic strategies and inspire the research and development of novel medications.

## Introduction

Breast cancer (BRCA) currently has the highest incidence rate among female tumours (1). In most patients, the disease is diagnosed in the early stages, and they show favorable prognosis following surgical resection of the primary tumours. However, once patients experience metastases, it will lead to the majority of BRCA-related deaths. As a type of regional metastasis, lymph node metastasis is less lethal than distant metastasis. However, it is the most common form of metastasis in BRCA patients, an important indicator affecting the efficacy of BRCA treatment, and a definite risk factor affecting long-term prognosis of individuals with BRCA (2).

The immune system is considered a decisive factor in cancer formation and progression (3). As the most important component of the human immune system, the lymphatic system performs the tasks of immunological surveillance and immune regulation (4). Moreover, the lymphatic circulatory system can regulate and maintain homeostasis and mediate lymphatic metastasis of tumours. Tumour-infiltrating lymphocytes (TILs) cross the boundary of oncology and immunology. TILs refer to immune cell populations infiltrating tumour tissues with high immune-related gene (IRG) expression, and a higher proportion of TILs is strongly associated with a higher survival rate of specific BRCA subtypes (5, 6). Several studies have reported TILs in tumours, including BRCA (7, 8), and increased proportions of TILs are associated with HER2 amplification, indicating prolonged survival (9). Increased proportions of TILs in BRCA tissue may suggest favorable responses to neoadjuvant therapy and have considerable predictive significance for adjuvant chemotherapy as well (10). With the development of bioinformatics (11, 12), researchers have started to quantify TILs and uncover personalised immune-related characteristics for the prognosis of different cancers by utilising the expression of IRGs (13–15). In particular, there have been several studies in BRCA that have developed prognostic models based on IRGs characteristics (16–19).

Accumulating evidence demonstrates that the immune system and IRGs might perform an important role in regulating BRCA patients’ treatment responses and long-term survival (7). Because IRGs are potentially correlated with lymphatic metastasis and the correlation between IRGs and lymph node metastatic prognosis in BRCA patients is yet to be systematically evaluated, we specifically selected patients with stage N+M0 BRCA from The Cancer Genome Atlas (TCGA) database, studied the expression profiles of IRGs and their predictive value using systematic bioinformatics analysis, and validated the prognostic model using a validation cohort from GSE20685 and Shandong Cancer Hospital and Institute (SCHI). Subsequently, the relevant regulatory axes in BRCA were explored. Our findings could add to the body of knowledge supporting prognostic biomarkers and treatment strategies for stage N+M0 BRCA.

## Materials and Methods

### Public Data Gathering and Processing

**Figure 1:**
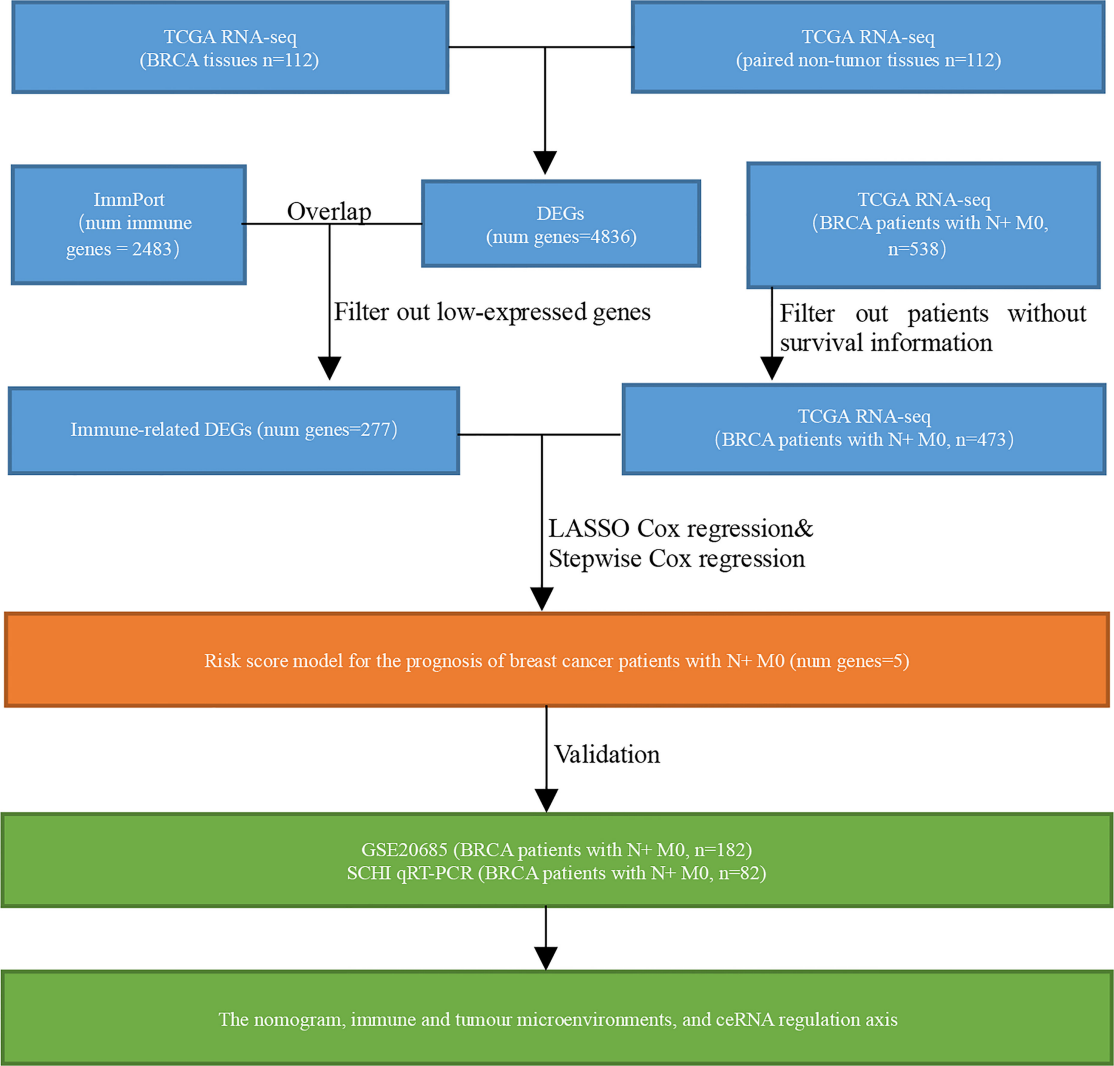
Flowchart.

The clinical, mutation, and RNA-seq information of 1222 BRCA cases was downloaded from TCGA database using the R package ‘TCGAbiolinks’. R packages ‘org.Hs.eg.db’ and ‘clusterProfiler’ were applied to annotate the IDs in RNA-seq data with Gene Symbol. The ‘merge’ function in R was used to precisely match and integrate the expression data with the clinical data by ID numbers. Finally, after excluding patients with missing survival information and cases with stage IV BRCA, data of 112 BRCA tissue samples, 112 paired paracancerous tissue samples, and 473 patients with stage N+M0 BRCA were obtained for subsequent analysis. The GSE20685 dataset was downloaded from GEO database (**Figure 1**).

### Training Cohort and Validation Cohort

TCGA training cohort included 473 cases with stage N+M0 BRCA, complete clinical and survival data, and tumour tissue gene expression data. It was used to explore and construct an IRG-based prognostic model.

The GSE20685 dataset contained RNA sequencing data from 327 primary BRCA patients, from which 182 patients with stage N+M0 BRCA were selected as the validation cohort.

The SCHI validation cohort included data of 82 patients from SCHI who had stage N+M0 BRCA and underwent surgical treatment between Jan 2012 and Dec 2014 (see **Table 1** and **Table S1** for more details). Total RNA was extracted from tumour tissue samples of this validation cohort to detect the expression of candidate IRGs and validate the performance of the prognostic model.

**Table 1:** Clinical features of the TCGA and SCHI cohorts.

Characteristics	TCGA cohort (n = 473)		SCHI cohort (n = 82)		*p*-value
	No.	Percentage (%)	No.	Percentage (%)	
Age					
≤ 60	293	61.9	68	82.9	0.000
> 60	180	38.1	14	17.1	
Living status					
Alive	450	95.1	75	91.5	0.274
Dead	23	4.9	7	8.5	
AJCC stage					
I-II	267	56.4	49	59.8	0.577
III	206	43.6	33	40.2	
T stage					
T1-2	379	80.1	72	87.8	0.100
T3-4	94	19.9	10	12.2	
N stage					
N1	309	65.3	53	64.6	0.903
N2-3	164	34.7	29	35.4	
Pharmaceutical treatment					
Yes	403	85.2	80	97.6	0.002
No	70	14.8	2	2.4	
Radiation treatment					
Yes	303	64.1	51	62.2	0.746
No	170	35.9	31	37.8	

### Identification of IRGs, Hub Genes, and Pathways

**Figure 2:**
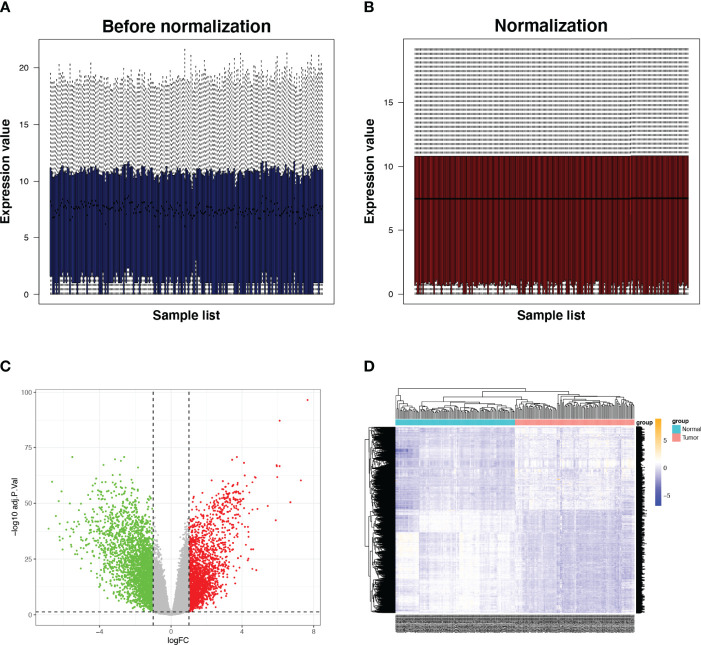
Expression of IRGs in BRCA. **(A, B)** Comparison before and after standardisation of expression data. **(C, D)** Volcano and heat maps of DEGs in tumor and normal tissues. Green dots represent genes that are down-regulated, red dots represent genes that are up-regulated and grey dots represent genes that are not significantly changed. IRG, immune-related gene; DEG, differentially expressed gene.

The RNA-seq data of 112 paired BRCA and paracancerous tissue samples were normalised utilising the R package ‘limma’ (**Figures 2A, B**). Parameter settings: |logFC|>1 and adjusted p value<0.05. Differentially expressed genes (DEGs) were defined as genes that were upregulated or downregulated in BRCA tissues. The volcanic maps and cluster heat maps (**Figures 2C, D**) were plotted using the R package ‘pheatmap’. The IRGs were downloaded from the ImmPort database. The intersection of DEGs and IRGs was taken, and genes with low abundance (i.e. genes whose original expression value is less than 15 in over 25% of all samples) were filtered to eventually obtain differentially expressed IRGs (**Figure 1**).

**Figure 3:**
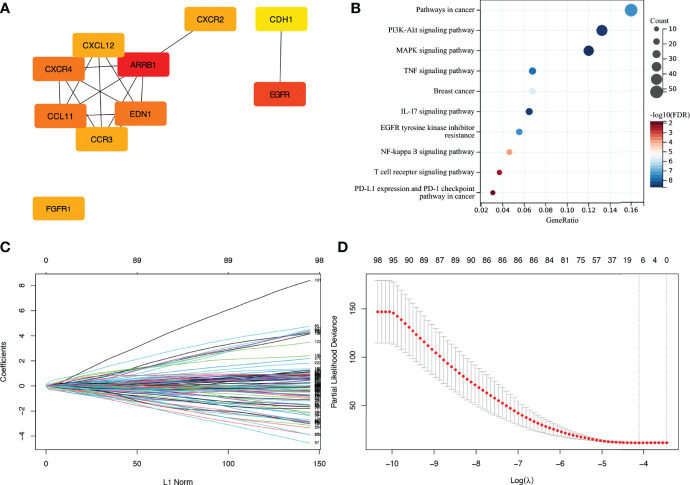
Enrichment analysis of IRGs and LASSO cox regression analysis. **(A)** Display of hub genes in IRGs. **(B)** Pathways for IRGs enrichment. **(C, D)** Coefficients and partial likelihood deviance of LASSO cox regression analysis. IRG, immune-related gene.

The interactive network analysis of IRGs was carried out using the String database. Next, for hub gene screening and regulated pathway enrichment analysis of IRGs, we utilised Cytoscape and DAVID database (**Figures 3A, B**).

### Calculation of Risk Scores

T_stage, N_stage, AJCC stage, pharmaceutical treatment, and radiation treatment were categorical variables. Age and risk score were considered continuous variables. Gene expression in TCGA cohort and GSE20685 cohort and ΔCt values of related genes in the SCHI cohort were continuous variables. X-tile 3.6.1 (20) was applied to estimate the best cutoff value for grouping IRGs into low or high expression groups. Hence, the continuous variables were converted into categorical variables to construct and validate the multigene prognostic model.

First, to discover prognosis-related IRGs in TCGA training cohort, a LASSO Cox regression analysis was performed by the R package ‘glmnet’ (**Figures 3C, D**). We then used Stepwise Cox regression analysis to identify a gene set that was most closely linked to the outcome of individuals with stage N+M0 BRCA. The sum of the product of the Cox regression coefficient and the expression value (high (1)/low (0) expression) for each gene was defined as the risk score. To split the patients into low- and high-risk groups, the median risk score was employed as a cutoff value. In the training and validation cohorts, Kaplan-Meier survival analysis was performed to test the prognostic differences between different scoring subgroups. To test if the risk score was an independent prognostic factor, a Cox multivariate regression analysis was performed by the R packages ‘survival’ and ‘survminer’. The area under the curve (AUC) was then determined to evaluate the accuracy of risk score prediction in each cohort using time-dependent receiver operating characteristic curve (ROC) analysis.

### Tumour Mutation Burden (TMB), Mutant-Allele Tumour Heterogeneity (MATH), and Nomogram

Using the R package ‘maftools’, we calculated the TMB and MATH values with mutation data of TCGA cohort and then combined these data with clinical data to plot a forest map of multivariate Cox hazard regression. The R package ‘rms’ was used to plot prognostic nomograms based on independent prognostic factors.

### Relationship Between Risk Scores and Immune Microenvironment, Tumour Microenvironment, and Immunotherapies

For immune-related analysis, we determined the infiltration scores of 24 immune cell types using the ImmuneCellAI database based on TCGA RNA-seq data and visualised the correlations of the risk score and all candidate IRGs with the proportions of infiltrating immune cells by the R package ‘corrplot’. The composition of stromal cells and immune cells was assessed by the R package ‘estimate’. We utilised the online tool Tumour Immune Dysfunction and Exclusion (TIDE) (21) to estimate patients’ response to immunotherapy of anti-CTLA4 and anti-PD1 to explore if there were differences in immunotherapy effectiveness between the two groups of patients.

### Construction of Competitive Endogenous RNA (ceRNA) Network

We built a ceRNA network to figure out what role IRGs might have in BRCA. Databases, such as ENCORI, miRTarBase, TargetScan, and TarBase, were used to predict miRNA targets that bound to IRGs. Based on the identified miRNAs, lncRNA targets interacting with the miRNAs were efficiently predicted using the ENCORI and LncBase databases. Finally, candidate lncRNA and miRNA expression and prognostic value were investigated.

### RT-qPCR Analysis

For the SCHI validation cohort, the total RNA extraction kit, DP439 (TIANGEN Biotech, Co., Ltd., Beijing, China), was used to isolate total RNA from tissue paraffin blocks of 82 patients with stage N+M0 BRCA containing tissues at the site of the primary lesion. A Prime Script RT reagent Kit with gDNA Eraser, RR047A (Takara Biomedical Technology, Co., Ltd., Japan), was used to synthesize cDNA from total RNA *via* reverse transcription in two steps. Then, the TB Green-based fluorescence quantitative PCR assay was performed using the Light Cycler 480 system (Roche, Switzerland). With ACTB as an internal reference gene, the relative expression value of a gene was obtained by calculating ΔCt as follows:

ΔCt (gene) = Ct (gene) - Ct (ACTB)

A higher ΔCt value is accompanied by a lower original expression value of a gene. The mRNA primers (**Table S2**) tested in this study were synthesised by General Biosystems, Co., Ltd. (Anhui, China).

### Statistical Analysis

SPSS version 26 for Mac and R version 4.0.3 for Mac were used for statistical analysis. The log-rank test was used to compare differences between KM curves. Quantitative data between groups were compared using the Wilcoxon test. The correlation between quantitative data between groups was expressed by Spearman’s coefficient. Statistical significance was defined as p < 0.05.

## Results

### IRGs, Hub Genes, and Pathway Enrichment Analysis

We identified 4836 DEGs in BRCA tissues, including 2675 downregulated genes and 2161 upregulated ones (**Figures 2C, D**). The intersection of 4836 DEGs and 2483 IRGs was filtered to obtain 423 IRGs. After excluding genes with low expression, a gene set consisting of 277 IRGs was finally obtained, among which *ARRB1*, *EGFR*, *CXCR4*, *CCL11*, *EDN1*, *EGFR1*, *CXCR2*, *CCR3*, *CXCL12*, and *CDH1* were hub genes. The major enriched pathways were PI3K-Akt, MARK, TNF, IL-17, NF-kappa B, EGFR tyrosine kinase inhibitor resistance, T cell receptor, and PD-L1 expression.

### Construction of an IRG-Based Prognostic Model

Seven IRGs whose expression was highly correlated with the outcome of patients with stage N+M0 BRCA in TCGA training cohort were identified using LASSO Cox regression analysis. These genes were *CDH1*, *FGF2*, *FGFR3*, *INHBA*, *IL33*, *S100B*, and *SCG2*. The coefficients and partial likelihood deviance are shown in **Figures 3C, D**. The above mentioned seven genes were then subjected to Stepwise Cox regression analysis to look for independent prognostic markers, and we finally developed a model incorporating the following five genes: *CDH1*, *FGFR3*, *INHBA*, *S100B*, and *SCG2* (**Table 2**). X-tile software was applied to figure out the appropriate cutoff value for the expression of the five genes based on the correlation between gene expression and overall survival (OS). Each gene was then defined to be in a low expression state (denoted by 0) or high expression state (denoted by 1) based on the cutoff value. Risk score = (-1.928) × *CDH1* status + (-1.641) × *FGFR3* status + 1.114 × *INHBA* status + (-1.871) × *S100B* status + 0.945 × *SCG2* status

**Table 2:** Cox regression analysis results.

Gene	B (coef)	sig.	Exp (B)
CDH1	-1.928	0.000	0.145 (0.053-0.400)
FGFR3	-1.641	0.001	0.194 (0.071-0.533)
INHBA	1.114	0.043	3.045 (1.035-8.963)
S100B	-1.871	0.000	0.154 (0.062-0.384)
SCG2	0.945	0.050	2.572 (1.002-6.606)

Subsequently, TCGA cohort was divided into a low-risk group (n = 248) and high-risk group (n = 225) using the median risk score (-3.799) as the cutoff value.

### Predictive Performance of the IRG-Based Prognostic Model

**Figure 4:**
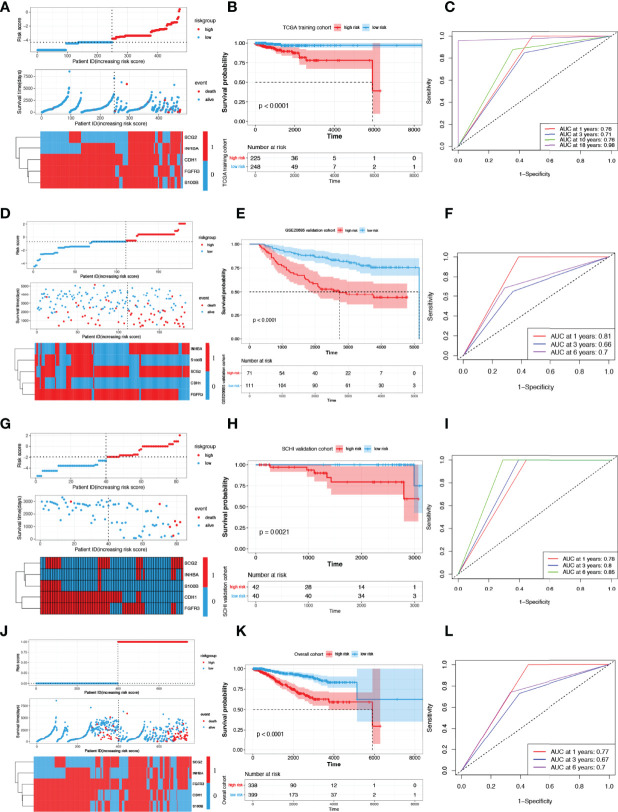
Construction and validation of risk scoring models. **(A)** Distribution of risk score, survival status, and the expression of five prognostic IRGs in TCGA training cohort. **(B, C)** OS curves for patients in different subgroups and AUCs in TCGA training cohort. Presentation of the above parameters in the GSE20685 validation cohort **(D–F)**, in the SCHI validation cohort **(G–I)** and in the overall cohort **(J–L)**. IRG, immune-related gene; AUC, area under the curve.

**Figure 4A** shows the risk score and survival status of each patient and the expression levels of the five candidate IRGs in TCGA cohort. The KM survival curve suggested that patients in the low-risk group had a significantly longer OS than those in the high-risk group (p < 0.0001, **Figure 4B**). The five IRG-based prognostic model predicted that the AUC values for 1-year, 3-year, 10-year, and 18-year postoperative survival rates were 0.76, 0.71, 0.76, and 0.98, respectively (**Figure 4C**).

**Figure 4D** shows the risk score and survival status of each patient and the expression levels of the five candidate IRGs in the GSE20685 validation cohort. The KM survival curve suggested that patients in the low-risk group had a significantly longer OS than those in the high-risk group (p < 0.0001, **Figure 4E**). The five IRG-based prognostic model predicted that the AUC values for 1-year, 3-year, and 6-year postoperative survival rates were 0.81, 0.66, and 0.70, respectively (**Figure 4F**).

**Figure 4G** shows the risk score and survival status of each patient and the expression levels of the five candidate IRGs in the SCHI validation cohort. The KM survival curve suggested that patients in the low-risk group had a significantly longer OS than those in the high-risk group (p = 0.0021, **Figure 4H**). The five IRG-based prognostic model predicted that the AUC values for 1-year, 3-year, and 6-year postoperative survival rates were 0.78, 0.80, and 0.85, respectively (**Figure 4I**). The results of the overall cohort are shown in **Figures 4J–L**.

### Independence Test and Subgroup Analysis

**Figure 5:**
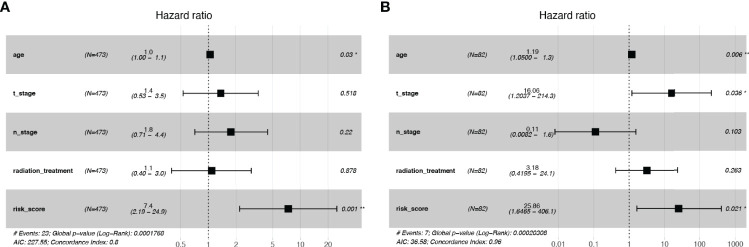
Independence tests for risk scores. **(A)** Multivariate Cox regression risk forest plot in TCGA training cohort. **(B)** Multivariate Cox regression risk forest plot in SCHI validation cohort.

We included clinicopathological parameters in TCGA cohort (age, N_stage, T_stage, and Radiation treatment) and plotted a forest map of multivariate hazard regression analysis: age (HR: 1.0; p = 0.03) and risk score (HR: 7.4; p = 0.001) were independent risk factors (**Figure 5A**). In the SCHI validation cohort, age (HR: 1.19; p = 0.006), T_stage (HR: 16.06; p = 0.036), and risk score (HR: 25.86; p = 0.021) were independent risk factors (**Figure 5B**).

**Figure 6:**
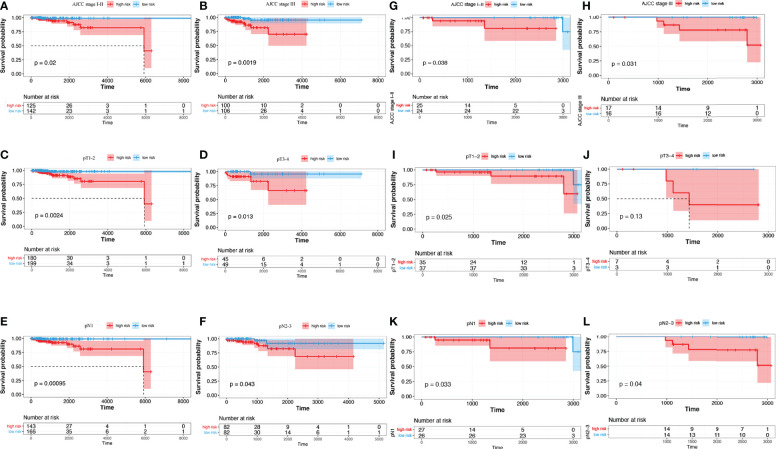
Subgroup analysis of risk score predictive ability. **(A–F)** TCGA training cohort. **(G–L)** SCHI validation cohort. **(A, G)** AJCC stage I-II. **(B, H)** AJCC stage III. **(C, I)** pT1-2. **(D, J)** pT3-4. **(E, K)** pN1. **(F, L)** pN2-3.

Subgroup analyses suggested that in TCGA cohort, for the AJCC stage subgroup, whether in the stage I-II subgroup (p = 0.02) or the stage III subgroup (p = 0.0019), the OS of low-risk patients was considerably longer than that of high-risk individuals (**Figures 6A, B**). For the T_stage subgroup, low-risk patients had a significantly longer OS than high-risk ones in both the T1-2 (p = 0.0024) and T3-4 subgroups (p = 0.013) (**Figures 6C, D**). Likewise, for the N_stage subgroup, low-risk patients had a significantly longer OS than high-risk ones in both the N1 (p = 0.00095) and N2-3 subgroups (p = 0.043) (**Figures 6E, F**). In the SCHI cohort, for the AJCC stage subgroup, whether in the stage I-II subgroup (p = 0.038) or the stage III subgroup (p = 0.031), the OS of low-risk patients was considerably longer than that of high-risk individuals (**Figures 6G, H**). For the T_stage subgroup, low-risk patients had a significantly longer OS than high-risk ones in the T1-2 subgroup (p = 0.025, **Figure 6I**); however, there was no statistically significant difference in the T3-4 subgroup (p = 0.13, **Figure 6J**). Likewise, for the N_stage subgroup, low-risk patients had a significantly longer OS than high risk ones in both the N1 (p = 0.033) and N2-3 subgroups (p = 0.04) (**Figures 6K, L**).

### Nomogram Establishment

**Figure 7:**
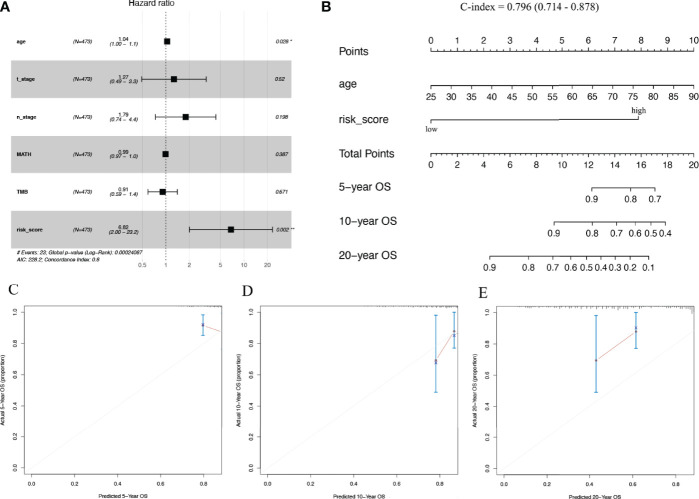
Predictive nomogram construction. **(A)** Hazard ratio and p‐value of the clinicopathological factors and risk scores involved in multivariate Cox regression in the TCGA cohort. **(B)** Nomogram to predict the 5-year, 10-year, and 20-year overall survival rate of patients with stage N+M0 BRCA. **(C–E)** Calibration curve for the nomogram. BRCA, breast cancer.

Multivariate cox regression risk forest plots for age, T_stage, N_stage, MATH, TMB, and risk score in the TCGA cohort showed that age (HR=1.04, p=0.028) and risk score (HR=6.82, p=0.002) were independent risk factors for OS (**Figure 7A**). We integrated the risk score with age to plot a nomogram to build a quantitative approach for OS prediction and it exhibited excellent predictive performance with its C-index being 0.796 (0.714–0.878) (**Figures 7B–E**).

### Correlation of IRG-Based Risk Scores With Immune Microenvironment and Clinical Characteristics

**Figure 8:**
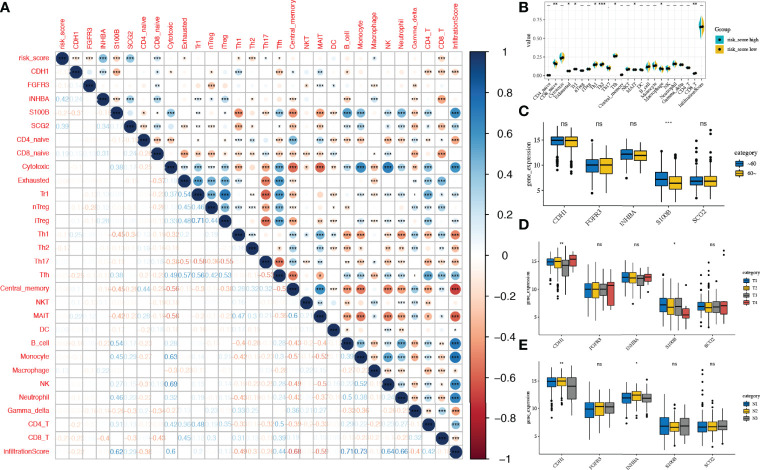
Correlation of risk scores with immune microenvironment and Clinical characteristics. **(A)** Correlation between the expression of IRGs and the proportions of immune cell infiltration. **(B)** Comparison of the proportions of immune cell infiltration between risk score subgroups. **(C–E)** Expression of the five candidate genes in different clinical subgroups (age, T-stage, N-stage) IRG, immune-related gene. *, **, *** and expanded form of "ns" denote p<0.05, p<0.01, p<0.001, and p≥0.05, respectively.

The correlation between immune infiltration and risk scores in patients with stage N+M0 BRCA was the first to be analysed. The results indicated that risk score was positively correlated with the proportion of infiltrated naive CD8+ T cells (r = 0.19, p < 0.001) and macrophages (r = 0.13, p < 0.05), but negatively correlated with the proportion of exhausted cells (r = -0.08, p < 0.05), Th1 cells (r = -0.11, p < 0.05), Th2 cells (r = -0.17, p < 0.001), Tfh cells (r = -0.07, p < 0.05), and CD8+ T cells (r = -0.21, p < 0.001), which had infiltrated (**Figures 8A, B**).

We next demonstrated a correlation between the expression of prognostic IRGs (*CDH1, FGFR3, INHBA, S100B, and SCG2*) and immune infiltration (**Figure 8A**). *CDH1* expression was found to be inversely associated with Tfh cell infiltration fraction (r = -0.25, p < 0.001). *FGFR3* expression correlated inversely with the abundance of nTreg (r = -0.28, p < 0.001). The expression level of *INHBA* was inversely correlated with the infiltration fraction of CD8+ T cells (r = -0.4, p < 0.001). *SCG2* expression correlated inversely with the infiltration fraction of Th1 cells (r = -0.34, p < 0.001). Additionally, *S100B* expression was found to be positively associated with B_cell infiltration fraction (r = 0.54, p < 0.001).

Next, we compared the expression of candidate IRGs in subgroups with different clinical characteristics, suggesting that *S100B* expression was considerably lower (p < 0.001) in elderly patients (>60 years); the expression of *CDH1* (p < 0.01) and *S100B* (p < 0.05) was correlated with T_stage. Furthermore, the expression of *CDH1* (p < 0.01) and *INHBA* (p < 0.05) in the N_stage subgroups was considerably different (**Figures 8C–E**)

### Risk Scores in the Prediction of Tumour Microenvironment and Immunotherapies

**Figure 9:**
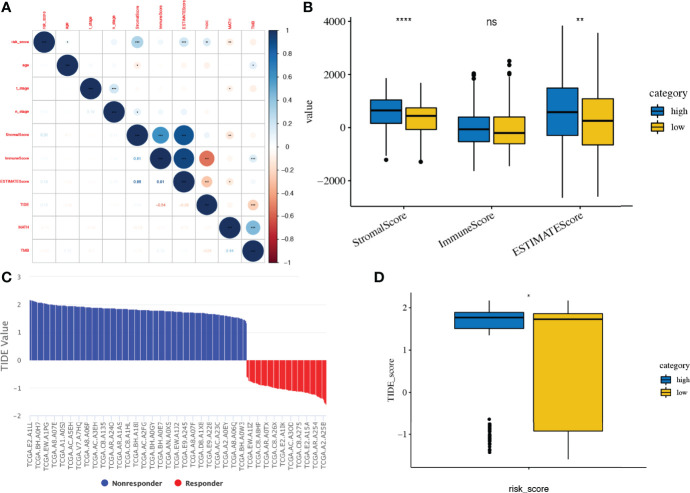
Tumour microenvironment and immunotherapy prediction. **(A, B)** Association of risk score and tumour microenvironment. **(C, D)** TIDE scores in different risk score subgroups. TIDE, Tumour Immune Dysfunction and Exclusion. *, **, ***, **** and expanded form of "ns" denote p<0.05, p<0.01, p<0.001, p<0.0001, and p≥0.05, respectively.

The matrix and immunological scores in the immune microenvironment were calculated using the ESTIMATE program. Although the risk score did not have a significant correlation with ImmuneScore, it did have a significant positive correlation with ESTIMATEScore (r = 0.19, p < 0.001) and StromalScore (r = 0.31, p < 0.001), and the low-risk score group had significantly lower ESTIMATEScore (p < 0.01) and StromalScore (p < 0.0001) than the high-risk score group (**Figures 9A, B**).

The response to immune checkpoint inhibitors was then assessed in subgroups based on risk scores, using the TIDE tool. Patients with a high-risk score had a significant higher TIDE score than those with low risk score (p < 0.05, **Figures 9C, D**).

### Construction of the lncRNA-miRNA-mRNA Network

**Figure 10:**
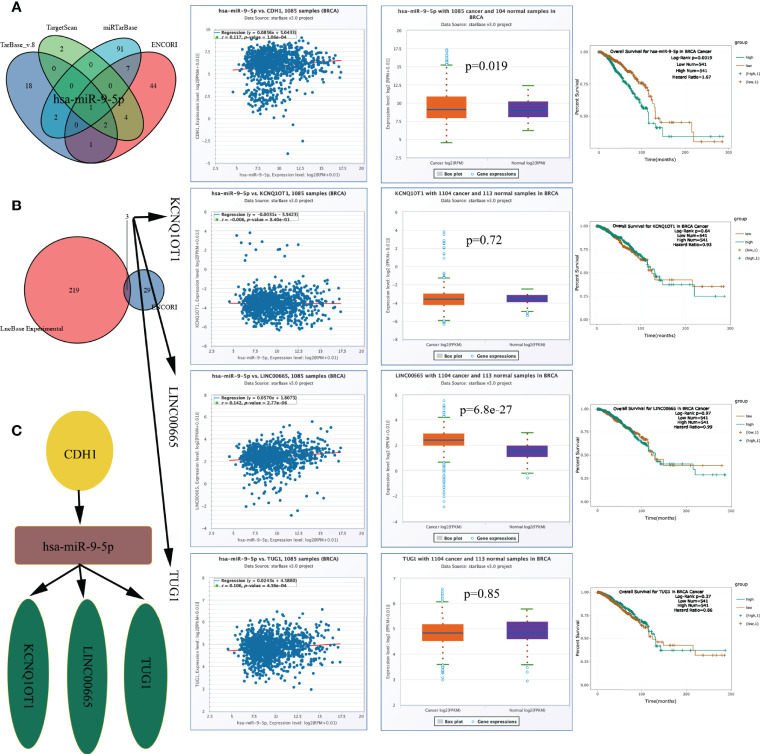
CeRNA network construction. **(A)** MiRNAs predicted by ENCORI, miRTarBase, TargetScan, and TarBase, and has-miR-9-5p expression level and prognostic value. **(B)** LncRNAs predicted by ENCORI and LncBase Experimental, and lncRNA KCNQ1OT1/LINC00665/TUG1 expression level and prognostic value. **(C)** The network of lncRNA–miRNA–mRNA. ceRNA, competing endogenous RNA.

As described above (**Figures 8D, E**), *CDH1* expression is correlated with both T_stage and N_stage. As a hub gene (**Figure 3A**), *CDH1* might play a crucial role in the progression of BRCA. To elucidate the potential molecular mechanisms of *CDH1* in BRCA, a lncRNA‒miRNA‒mRNA interaction network was constructed. miR-9-5p was identified as a targeted miRNA that binds to *CDH1* based on ENCORI, miRTarBase, TargetScan, and TarBase data (**Figure 10A**). *CDH1* expression correlated positively with the expression level of miR-9-5p in TCGA BRCA cohort (r = 0.117, p = 1.06e-04, **Figure 10A**). miR-9-5p was upregulated in the tumour samples (p = 0.019), and the BRCA patients with high miR-9-5p levels had significantly shorter OS (p = 0.0019) (**Figure 10A**).

We constructed a lncRNA-miRNA-mRNA axis based on the outcome above and its upstream lncRNA targets. The lncRNAs KCNQ1OT1, LINC00665, and TUG1 were defined as targets, and the expression level of miR-9-5p was positively correlated with those of LINC00665 (r = 0.142, p = 2.77e-06) and TUG1 (r = 0.106, p = 4.56e-04) (**Figure 10B**). LINC00665 was up-egulated in the tumour samples (p = 6.8e-27), the expression levels of the above lncRNAs did not correlate with OS (**Figure 10B**). **Figure 10C** demonstrates the ceRNA network. The regulatory axis of the lncRNA KCNQ1OT1-LINC00665-TUG1/miR-9-5p/CDH1 may be crucial in the evolution of BRCA.

## Discussion

The occurrence and development of many types of malignancies are associated with disorders of the immune system. IRG activation has been shown in some studies to diminish BRCA recurrence in patients with BRCA (22). Previous studies have shown that immune-related prognostic markers are mostly associated with TILs (23–25). With the development of bioinformatics in recent years, we can now quantify TILs by determining gene expression. In BRCA, models based on TILs have been developed to predict patient prognosis and drug efficacy (26–29). However, the role of IRGs in N+M0 BRCA before this study was unclear; hence, we investigated the role of IRGs in N+M0 BRCA. We first elucidated the expression of IRGs in N+M0 BRCA. We identified 423 differentially expressed IRGs. We constructed an IRG prognostic risk scoring model, according to RNA-seq and clinical data of patients with stage N+M0 BRCA in TCGA, and it enabled us to accurately differentiate patient prognosis based on these criteria. Immunotherapy may be more beneficial to those with a low-risk score. Immune cell infiltration and tumour microenvironment differed significantly between the two risk score subgroups. Age and IRG-based risk score were independent factors affecting N+M0 BRCA patient prognosis. These factors were included in a prognosis nomogram. We also constructed a regulatory axis of the ceRNA network to explore the mechanism of the progression of BRCA.

We first identified the differentially expressed IRGs from breast cancer tissues versus paired normal tissues in the TCGA database, and we considered that screening for DEGs between such paired samples might reduce the bias from individual differences. The protein-protein interaction analysis suggested that *CDH1* is a hub gene. Apart from PI3K-Akt and MARK, differentially expressed IRGs were mainly enriched in pathways associated with inflammatory responses and immune responses, including IL-17, T cell receptor, NF-kappa B, and PD-L1 expression. These results indicate that cellular immunity may have a significant impact on the occurrence and progression of BRCA. As a cancer suppressor gene coding for E-cadherin and a type of calcium-dependent cell adhesion protein, *CDH1* plays a role in regulating cell adhesion, proliferation, and migration. Hence, *CDH1* dysfunction can promote certain biological behaviours of cells, such as invasion and metastasis. Mutation of this gene is closely associated with BRCA, rectal cancer, and other cancer types, and the expression of *CDH1* is downregulated in various tumour tissues (30, 31). Jian et al. (32) confirmed that methylation in the *CDH1* gene promoter region might cause a reduction in *CDH1* expression, and BRCA patients with lower *CDH1* expression were more prone to lymph node metastasis and experienced lower OS rate and shorter disease-free survival. The protein encoded by the FGFR3 gene corresponds to an important component of the fibroblast growth factor receptor family that binds to fibroblast growth factor and plays a key role during overall skeletal maintenance as well as development. Aberrant expression of FGFR3 is also commonly observed in various cancers and can directly or indirectly activate various downstream signalling pathways, such as the FGFR3 signalling pathway (33), PI3K-AKT signalling pathway (34), and RAS/RAF/MEK/MAPK pathway (35), which are key mediators of malignant tumorigenesis and progression. According to TCGA and GTEx data, FGFR3 is significantly upregulated in breast tumours. In addition, FGFR3 activation can make BRCA cells less sensitive to drugs, such as fulvestrant and tamoxifen (36), and thus FGFR3 can be a candidate therapeutic target (37). S100B encodes S100 calcium-binding protein B, a molecule associated with tumour metastasis and progression, which regulates the proliferation and metabolism of cancer cells through physical interactions with other molecules. For example, overexpression of S100B leads to enhanced migration and invasion of lung cancer cell lines, thereby promoting brain metastasis (38). Serum S100B and S100B autoantibodies are biomarkers of lung cancer brain metastasis (39). In melanoma, serum S100B concentration was positively correlated with tumour stage and negatively correlated with survival rate (40). Furthermore, elevated serum S100B levels were associated with melanoma metastasis, and lower serum S100B levels were associated with improved survival (41). In the present study, S100B was downregulated in BRCA tissues, which was also corroborated by Yen et al. (42), who also noted that a higher S100B expression predicted good OS in ER-negative BRCA patients and longer metastasis-free survival in all BRCA patients. The expression levels of INHBA and SCG2 in this model were negatively correlated with OS in BRCA patients, and the INHBA gene encodes a member of the transforming growth factor beta superfamily. This gene is significantly overexpressed in BRCA tissues and its high expression status in a variety of tumours, such as colon cancer (43), oesophageal squamous cell carcinoma (44), lung adenocarcinoma (45), and bladder and uroepithelial carcinoma (46), suggests a poor prognostic outcome. SCG2 is a member of neuroendocrine proteins, whose important functions include promoting neointima formation and enhancing endothelial angiogenesis (47). Cury et al. (48) found that SCG2 could be an important indicator to differentiate the progression and prognosis of NSCLC patients.

The expression of five IRGs was subsequently used to build a prognostic risk score model. Similar attempts have been made in studies on tumours, such as BRCA, cervical cancer, osteosarcoma, and Ewing sarcoma (16, 18, 19, 49–54). For example, Zhu et al. (16) and Zhao et al. (18) constructed 12- and 27-gene models of IRGs to predict the prognosis of BRCA patients, respectively. Wang et al. (49) constructed a 5-gene IRG prognostic model for triple-negative breast cancer (TNBC) patients, while Yang et al. (50) combined both hypoxia and immune genes to construct a 6-gene composite prognostic model for the triple-negative subgroup. Tian et al. (19) used an 8-gene IRG model to predict BRCA recurrence, and Tan et al. (51) used a 9-gene IRG model to predict preoperative axillary lymph node metastasis in TNBC. Compared to the above studies, we made the first attempt in N+M0 stage BRCA, and the number of included IRGs was minimal, while ensuring the predictive efficacy of the model, which allowed for a more simplified model. S100B and SCG2 have been reported in the above models, while CDH1, FGFR3, and INHBA are specific to this cohort, which may be related to the characteristics of patients with stage N+M0 BRCA and to the fact that we filtered out some low-expressed genes to facilitate model application and promotion. We also validated the performance of this model using the training and validation cohorts. The results indicated considerable statistical differences in OS between the different risk score subgroups of stage N+M0 BRCA patients and that high risk scores resulted in a higher incidence of patient fatalities. Although the SCHI validation cohort was followed up for 3329 days at most (markedly less than the maximum 8556 days of follow-up for TCGA training cohort), the prognostic model satisfactorily predicted the 1-year, 3-year, and 6-year OS. We also intend to validate the long-term prognostic performance of the model with 10-year or even 20-year follow-up data. Risk score was found to be an independent prognostic factor for individuals with stage N+M0 BRCA in multivariate COX regression analyses. Subgroup analyses suggested that regardless of clinical staging (AJCC stage I-II vs. AJCC stage III), tumour size (pT1-2 vs. pT3-4) or the number of lymph node metastases (pN1 vs. pN2-3), risk score was always an effective tool for forecasting the OS of patients with stage N+M0 BRCA. To further improve the predictive performance, we used mutation data of TCGA cohort to calculate the TMB and MATH of each patient with stage N+M0 BRCA. Previous research has found that cancer patients with high TMB have a better survival (55–57), and MATH is a new approach to characterize intra-tumour heterogeneity. Multiple studies have suggested that high MATH values are correlated with poor cancer prognosis (58–60). Unfortunately, TMB and MATH were not shown to be independent predictive factors in the study; therefore, we integrated age and risk score into the plotted nomogram. The model performed satisfactorily in predicting the 5-year, 10-year, and even 20-year OS, according to the validation results.

Another major finding is that the proportion of TILs in tumour tissue correlated significantly with the expression levels of the five IRGs in the model. In addition, the relationship between risk score and tumour microenvironment further confirmed the role of IRGs in the tumour microenvironment. Immune checkpoint inhibitors may benefit individuals with malignant tumours, including metastatic BRCA, and immunotherapy is becoming a novel treatment option. The level of immune checkpoint molecules (e.g. CTLA4, PD-L1, and PD-1) has been used as a biomarker for predicting immunotherapy efficacy (61, 62). TIDE is a new algorithm that efficiently predicts the efficiency of immune checkpoint inhibitors by combining the two mechanisms of tumour immune escape (rejection reaction and immune dysfunction), outperforming single markers (21). Given the above, we evaluated the efficacy of immunotherapies, demonstrating that immunotherapies may be more beneficial to patients with low risk scores.

When analysing the relationship between IRGs and clinical staging, we noticed that *CDH1*, as a hub gene, has a correlation with both T_stage and N_stage; its coefficient in the model was up to 1.928, suggesting that *CDH1* contributed most to the risk score and might be closely associated with the progression of BRCA. To explore the mechanism by which *CDH1* regulates BRCA progression, we constructed a lncRNA-miRNA-mRNA network and established a lncRNA KCNQ1OT1-LINC00665-TUG1/miR-9-5p/CDH1 regulatory axis after strict filtering. Bandini et al. (63) suggested that miR-9-5p can inhibit BRCA cell proliferation by negatively regulating the androgen receptor, whereas TUG1 can regulate EIF5A2 expression by endogenous competition with miR-9-5P, thereby regulating the sensitivity of BRCA cells to doxorubicin (64). Moreover, *via* modulating miR-379-5p/LIN28B, LINC00665 can promote BRCA cell proliferation, invasion, and migration (65). miR-107 can be regulated by lowering the expression of KCNQ1OT1 to inhibit BRCA cell proliferation and migration (66). Furthermore, we discovered that miR-9-5p correlated with the prognosis of BRCA patients. These lines of evidence suggest that the lncRNA KCNQ1OT1-LINC00665-TUG1/miR-9-5p/CDH1 regulatory axis might play a crucial role in BRCA development. Therefore, additional research is required to validate this finding.

This study has several limitations. First, given that BRCA is a typical multigenic disease, the constructed prognostic model based only on IRGs has an inherent bias. Second, because the clinical data of TCGA training cohort did not include data on molecular subtyping, further studies need to be conducted to confirm whether the predictive performance of the model changes with different molecular subtypes. Finally, the regulatory axis of the ceRNA network needs to be validated *in vitro* and *in vivo*, and we are currently conducting studies on the mechanisms involved in the effects of miR-9-5p expression levels on the development and prognosis of breast cancer.

In summary, we developed a five-IRG prognostic scoring model *via* comprehensive and systemic bioinformatics investigation. The results of the SCHI validation cohort suggested that regardless of clinical staging, tumour size, or the number of lymph node metastases, this model demonstrated good predictive performance in forecasting the prognosis of patients with stage N+M0 BRCA and can determine the sensitivity of patients to immunotherapies, which will be conducive for developing personalised therapeutic strategies and inspiring the research and development of new medications. Our study results also confirmed that the lncRNA KCNQ1OT1-LINC00665-TUG1/miR-9-5p/CDH1 regulatory axis might play an essential role in BRCA progression. Further studies are required to verify these results.

## Data Availability Statement

The original contributions presented in the study are included in the article/**Supplementary Material**. Further inquiries can be directed to the corresponding authors.

## Ethics Statement

The studies involving human participants were reviewed and approved by The institutional review board of Shandong Cancer Hospital and Institute. The ethics committee waived the requirement of written informed consent for participation.

## Author Contributions

CT collected the patient’s clinicopathological data and downloaded external data sets. CT followed up on the patient’s survival information, performed qRT-PCR experiments on the SCHI cohort, and prepared the manuscript. XS and YW supervised the research and revised the manuscript. All authors contributed to the article and approved the submitted version.

## Conflict of Interest

The authors declare that the research was conducted in the absence of any commercial or financial relationships that could be construed as a potential conflict of interest.

## Publisher’s Note

All claims expressed in this article are solely those of the authors and do not necessarily represent those of their affiliated organizations, or those of the publisher, the editors and the reviewers. Any product that may be evaluated in this article, or claim that may be made by its manufacturer, is not guaranteed or endorsed by the publisher.
