# Supplementary material for: Prognostic Characteristics of Immune-Related Genes and the Related Regulatory Axis in Patients With Stage N+M0 Breast Cancer
Source: Front Oncol. 2022 Jun 16;12:878219. doi: 10.3389/fonc.2022.878219 (PMC9243266; doi:10.3389/fonc.2022.878219)
Supplement: Supplementary file 2 [file Table_2.docx]

Table 2. Primer sequences of five genes tested in this study.

| Gene | Forward 5’ -3’ | Reverse 5’ -3’ |
| --- | --- | --- |
| CDH1 | CTGCCAACTGGCTGGAGATT | CTGGAGAACCATTGTCTGTAGC |
| FGFR3 | TGCGTCGTGGAGAACAAGTTT | GCACGGTAACGTAGGGTGTG |
| INHBA | GAAGAGACCCGATGTCACCC | TGACTCGGCAAACGTGATGA |
| S100B | GGAGACGGCGAATGTGACTT | TCAAAGAACTCGTGGCAGGC |
| SCG2 | TGAGATGAAACGCTCAGGGC | GCCCATTCTGTAACCTCCCA |
